# Situación de los laboratorios públicos productores de antivenenos en América Latina

**DOI:** 10.26633/RPSP.2019.92

**Published:** 2019-11-19

**Authors:** Hui Wen Fan, Marco Antonio Natal Vigilato, Julio Cesar Augusto Pompei, José María Gutiérrez, en representación de la Red de Laboratorios Públicos Productores de Antivenenos de América Latina (RELAPA)

**Affiliations:** 1 Instituto Butantan Instituto Butantan São Paulo Brazil Instituto Butantan, São Paulo. Brazil; 2 Centro Panamericano de Fiebre Aftosa Organización Panamericana de la Salud Rio de Janeiro Brasil Centro Panamericano de Fiebre Aftosa, Organización Panamericana de la Salud, Rio de Janeiro. Brasil.; 3 Instituto Clodomiro Picado, Facultad de Microbiología Universidad de Costa Rica San José Costa Rica Instituto Clodomiro Picado, Facultad de Microbiología, Universidad de Costa Rica, San José, Costa Rica; 4 Los autores de la RELAPA y sus afiliaciones se mencionan al final del manuscrito. Los autores de la RELAPA y sus afiliaciones se mencionan al final del manuscrito. Los autores de la RELAPA y sus afiliaciones se mencionan al final del manuscrito.

**Keywords:** Antivenenos, servicios laboratoriales de salud pública, animales venenosos, América Latina, Antivenins, public health laboratory, animals, poisonous, Latin America, Antivenenos, serviços laboratorias de saúde pública, animais venenosos, América Latina

## Abstract

Se analiza la situación de los laboratorios públicos productores de antivenenos en América Latina, con base a los resultados de en un taller coordinado por el Centro Panamericano de Fiebre Aftosa (PANAFTOSA) de la Organización Panamericana de la Salud (OPS). Nueve países en la región poseen doce laboratorios públicos que producen y distribuyen antivenenos contra venenos de diferentes animales ponzoñosos. Se discutió la situación de cada laboratorio, se analizó el escenario actual caracterizado por las crecientes demandas regulatorias y la heterogeneidad de estos en términos de infraestructura y capacidad productiva y se planteó la necesidad de concertar procesos de cooperación regional dirigidos a mejorar la disponibilidad de antivenenos, incluyendo proyectos de investigación y desarrollo para el mejoramiento de los procesos y las tecnologías; estudios del perfil de la capacidad neutralizante de los antivenenos contra diferentes venenos, y programas de capacitación técnica de profesionales y personal técnico. En el contexto actual, en el que la Organización Mundial de la Salud elaboró una estrategia global para la prevención y el control de los envenenamientos ofídicos, el Centro PANAFTOSA de la OPS ha asumido la coordinación de estas acciones en las Américas, mejorar la disponibilidad de antivenenos es prioritaria. Como resultado de ese taller, se creó la Red de Laboratorios Públicos Productores de Antivenenos de América Latina (RELAPA), con el objetivo de fortalecer estos laboratorios y de aumentar la disponibilidad y accesibilidad de antivenenos eficaces y seguros a toda América Latina.

## Abstract (en)

The situation of public laboratories manufacturing antivenoms in Latin America was analyzed, based on the results of a workshop coordinated by the Pan American Foot-and-Mouth Disease Center (PANAFTOSA) of the Pan American Health Organization (PAHO). Nine countries in the region have 12 public laboratories that produce and distribute antivenoms for use against different venomous animals. The situation of each laboratory was discussed, and an analysis was conducted of the current scenario, which is characterized by increasing regulatory requirements that vary in terms of infrastructure and production capacity. The authors identified a need to organize regional cooperation processes to improve the availability of antivenoms, including: research and development projects to improve processes and technologies; studies of the capacity of antivenoms to neutralize different poisons; and technical training programs for professionals and technical personnel. In the current context, in which the World Health Organization has prepared a global strategy for the prevention and control of snakebite envenoming, PANAFTOSA has taken on coordination of this initiative in the Americas. Improving the availability of antivenoms is the priority. As a result of the workshop, the RELAPA network was created, bringing together public laboratories that manufacture antivenoms in Latin America, in order to strengthen these laboratories and increase the availability of, and access to effective and safe antivenoms throughout Latin America.

## RESUMO

A situação dos laboratórios públicos produtores de soros antivenenos na América Latina foi analisada com base nas conclusões de um seminário coordenado pelo Centro Pan-Americano de Febre Aftosa (PANAFTOSA) da Organização Pan-Americana da Saúde (OPAS). Em nove países da Região, encontram-se 12 laboratórios públicos que produzem e distribuem soros contra venenos de diferentes animais peçonhentos. Examinou-se a situação de cada laboratório, analisou-se o panorama atual marcado por crescentes demandas de regulação e pela heterogeneidade da infraestrutura e capacidade produtiva dos laboratórios e destacou-se a necessidade de um esforço concertado de cooperação regional com vistas a aumentar a disponibilidade de soros antivenenos, englobando projetos de pesquisa e desenvolvimento para o avanço dos processos e tecnologias, estudos do perfil da capacidade de neutralização dos soros contra diversos venenos e programas de capacitação técnica de profissionais e pessoal técnico. No contexto atual, em que uma estratégia mundial de prevenção e controle de acidentes ofídicos foi elaborada pela Organização Mundial da Saúde e que a coordenação das ações relacionadas na Região das Américas foi assumida pelo PANAFTOSA, melhorar a disponibilidade de soros antivenenos é prioridade. Como resultado deste seminário, a Rede de Laboratórios Públicos Produtores de Soros Antivenenos da América Latina (RELAPA) foi formada com o objetivo de fortalecer os laboratórios e aumentar a disponibilidade e a acessibilidade de soros antivenenos eficazes e seguros em toda América Latina.

Los envenenamientos por mordeduras y picaduras de animales ponzoñosos constituyen un serio problema de salud pública en América Latina (1–4). Se estima que en el continente americano ocurren cada año alrededor de 57 500 casos de envenenamientos por mordeduras de serpiente (5). En cuanto a envenenamientos por picadura de escorpiones, el número de casos es mayor ya que solo en Brasil y México se reportan aproximadamente 120 mil y 300 mil casos cada año, respectivamente (6,7). Los envenenamientos por picadura de araña de los géneros *Loxosceles, Latrodectus* y *Phoneutria* también son de relevancia en algunos países de región, en Brasil, Argentina y México se reportan más de 20 mil casos cada año (6, 8–11). Aunque se carece de datos exactos, se ha notado un aumento en los reportes de accidentes con orugas de lepidópteros del género *Lonomia* en varios países de América Latina (12–14).

Algunos de los envenenamientos son tratados de manera sintomática, sin embargo, en general, la terapia se basa en la administración intravenosa de antivenenos obtenidos a partir del plasma sanguíneo de equinos inmunizados con venenos de estos animales (15). América Latina cuenta con un conglomerado de laboratorios productores de antivenenos tanto en el ámbito público como privado, que abastecen de estos productos a la mayoría de los países de la región (16).

La carga que los envenenamientos imponen a los sistemas de salud está relacionada con la morbilidad y mortalidad resultante de las complicaciones locales y sistémicas, tales como necrosis muscular, hemorragias, infección generalizada, lesión renal y colapso cardiocirculatorio, muchas veces relacionadas con el retraso en la terapia especifica con antivenenos y con el tratamiento de soporte (4, 5). Las secuelas y discapacidades provocadas sobre todo por los envenenamientos por mordeduras de serpientes, además de representar un problema de salud, tienen implicaciones sociales y económicas, por la pérdida de condiciones laborales para los pacientes afectados (2).

Reconociendo el impacto de los envenenamientos en grupos de población vulnerables a nivel global, la Organización Mundial de la Salud (OMS) incluyó en el 2017 el ofidismo en la lista de enfermedades tropicales desatendidas. En mayo del 2018, la Asamblea Mundial de la Salud, reunida en Ginebra, aprobó una resolución sobre el tema de los envenenamientos por mordeduras de serpiente, en la cual se urge a los estados miembros a tomar una serie de acciones para reducir el impacto de los mismos (17). Algunas de estas acciones se enfocan en el incremento de la disponibilidad y accesibilidad de antivenenos eficaces y seguros, hecho que está directamente relacionado con el mejoramiento de los laboratorios productores, incluyendo la implementación de Buenas Prácticas de Manufactura (BPM) y la meticulosa evaluación preclínica y clínica de los productos. En seguimiento de esta resolución de la Asamblea Mundial de la Salud, la OMS presentó recientemente un plan estratégico para la prevención y el control de los envenenamientos por mordeduras de serpiente (18). La Organización Panamericana de la Salud (OPS), a través del Centro Panamericano de Fiebre Aftosa (PANAFTOSA), ha iniciado un programa de acción en la región en este tema, que se suma a sus actividades relacionadas con el control de la fiebre aftosa, las zoonosis y la inocuidad de los alimentos.

El objetivo de este artículo es presentar la situación de los productores de antivenenos en América Latina y los procesos de cooperación regional dirigidos a mejorar la disponibilidad de antivenenos en la Región de las Américas.

## MATERIALES Y MÉTODOS

Del 23 al 25 de octubre del 2018 se efectuó en el Instituto Butantan, en São Paulo, Brasil, un taller regional, coordinado por PANAFTOSA, con la participación de representantes de doce laboratorios públicos productores de antivenenos de Argentina, Bolivia, Brasil, Colombia, Costa Rica, Ecuador, México, Perú y Venezuela. Se identificaron los laboratorios activos o con reciente producción y distribución de antivenenos a nivel nacional. Una guía fue previamente enviada para preparar las presentaciones de cada uno de los laboratorios, conteniendo: datos generales del laboratorio, aspectos técnicos básicos de la producción (incluyendo cantidades producidas), situación ante el ente regulador del país, principales problemas técnicos del laboratorio, y necesidades de cooperación y apoyo técnico. En el caso de laboratorios con actividades interrumpidas, se preguntó cuándo ocurrió la interrupción, las razones, las cantidades producidas antes de la interrupción, y el planteamiento con respecto a retomar la producción. Con base en los datos suministrados en las presentaciones y en las discusiones efectuadas, este trabajo presenta los principales aspectos tratados en dicho taller.

## RESULTADOS Y DISCUSIÓN

A continuación, se describe la situación actual de los laboratorios productores en cada país.

**Argentina:** Los antivenenos son producidos por el Instituto Nacional de Producción de Biológicos, una de las once unidades técnico-operativas de la Administración Nacional de Laboratorios e Institutos de Salud-ANLIS “Dr Carlos Malbrán”, organismo dependiente de la Secretaría de Gobierno de Salud de la Nación. Cuenta con un sistema de gestión de calidad basado en la norma ISO 9001:2008. El portafolio de productos incluye antivenenos generados en caballos contra venenos de serpientes, arañas y escorpión. Estos productos se usan casi exclusivamente en la República Argentina. El Instituto cuenta con la autorización para producir antivenenos, otorgado por el ente regulador del gobierno argentino ANMAT (Administración Nacional de Medicamentos, Alimentos y Tecnologías Médicas).

**Bolivia:** El Laboratorio Productor de Antiveninas, perteneciente al Instituto Nacional de Laboratorios de Salud (INLASA), es el responsable de la producción de antivenenos en el Estado Plurinacional de Bolivia. Inició la producción en el año 2000 y actualmente el laboratorio produce antivenenos antiofídicos contra venenos de vipéridos y un antiveneno anti-*Latrodectus*, todos generados en burros. Estos antivenenos se utilizan exclusivamente en Bolivia.

**Brasil:** Existen cuatro laboratorios públicos de producción de antivenenos en Brasil y un laboratorio nacional para el control de calidad de los mismos, el Instituto Nacional de Controle de Qualidade em Saúde. Todos los antivenenos manufacturados en Brasil están estandarizados para tener la misma potencia neutralizante. Existe una coordinación estrecha entre los cuatro laboratorios productores y el Ministerio de Salud para que haya más de un productor para los antivenenos y otras inmunoglobulinas equinas de mayor demanda en el país. A continuación, se describen las actividades de los laboratorios en Brasil:

*Instituto Butantan.* Es la institución con mayor volumen de producción de antivenenos en Brasil, produce inmunoglobulinas de origen animal y vacunas desde 1901. Depende de la Secretaría de Salud del gobierno del Estado de São Paulo, actualmente manufactura 8 tipos diferentes de antivenenos contra venenos de diferentes especies animales; la Agência Nacional de Vigilância Sanitária (ANVISA), que es el agente regulador nacional le ha otorgado el certificado de BPM y efectúa regularmente inspecciones de sus instalaciones y procesos. Sus antivenenos se utilizan fundamentalmente en Brasil, aunque en ocasiones, por solicitud de autoridades de salud de otros países de la Región, se han efectuado donaciones de antivenenos, en algunos casos por intermedio de la OPS.

*Fundação Ezequiel Dias (FUNED).* Fundada en 1907, actualmente es una institución dependiente de la Secretaría de Salud del gobierno del Estado de Minas Gerais. Produce varios tipos de antivenenos contra venenos de serpientes y escorpiones. En el 2017 se inició la adecuación de la planta de producción y los procesos a las BPM, incluyendo la infraestructura, el sistema de agua, los sistemas de aire y los equipos. Se planea una nueva inspección de ANVISA para retomar la producción de antivenenos durante el año 2019.

*Instituto Vital Brazil.* Creado en 1919, está vinculado a la Secretaría de Salud del gobierno del Estado de Rio de Janeiro. Produce cinco tipos de antivenenos contra venenos de serpientes, antiveneno anti-escorpiónico y un antiveneno anti-arañas. Recientemente desarrolló un antiveneno contra veneno de abeja, el cual está siendo evaluado a nivel clínico (fase III). Actualmente se están realizando remodelaciones en algunos puntos de la infraestructura de los laboratorios, como respuesta a las inspecciones de ANVISA.

*Centro de Produção e Pesquisa de Imunobiológicos (CPPI).* Es una institución dependiente de la Secretaría de Salud del gobierno del Estado de Paraná. Inició la producción de antivenenos en 1987 y hasta el año 2014 produjo dos tipos de antivenenos. Entre los años 2014 y 2016 participó en la modalidad de producción compartida con otros institutos productores de Brasil. Actualmente se plantea un proyecto en el CPPI para la construcción de una nueva planta de producción, la cual se prevé esté construida para el año 2022.

**Colombia:** La producción de antivenenos en el sector público es efectuada en el Instituto Nacional de Salud (INS), a través de su Dirección de Producción. Este laboratorio produce antivenenos contra venenos de serpientes y contra veneno de *Lonomia* sp. En el 2018 fueron producidos cerca de 25 000 viales. Cuenta con certificado de BPM expedido por el Instituto Nacional de Vigilancia de Medicamentos y Alimentos (INVIMA).

**Costa Rica:** El Instituto Clodomiro Picado, perteneciente a la Universidad de Costa Rica, fue fundado en 1970. Produce un antiveneno polivalente, para tratar envenenamientos por especies de la familia Viperidae, el cual se distribuye en Costa Rica, Panamá, Nicaragua, Honduras, El Salvador, Guatemala, Belice, Ecuador y la isla caribeña de Santa Lucía. Produce además un antiveneno anti-*Micurus* y un antiveneno poliespecífico contra venenos de serpientes del África subsahariana (EchiTAb-plus-ICP). El Instituto cuenta con un sistema de gestión de calidad basado en la norma ISO 9001:2015 y con un certificado de BPM otorgado por el Ministerio de Salud de Costa Rica.

**Ecuador:** La producción de antivenenos para el tratamiento de envenenamientos por mordeduras de serpiente se inició en el Instituto Nacional de Higiene y Medicina Tropical “Dr Leopoldo Izquieta Pérez” en 1981. En el año 2012, el Ministerio de Salud Pública escindió ese Instituto, creando el Instituto Nacional de Salud Pública e Investigaciones (INSPI), que continuó a cargo de la producción de antivenenos. Entre los años 2007 y 2012 se produjo un volumen promedio de 7 000 frascos por año de antiveneno contra venenos de serpientes. La producción fue suspendida el año 2014 por parte del ente regulador por asuntos relacionados con BPM. En años recientes el país ha importado los antivenenos requeridos por su sistema de salud. Recientemente se ha presentado un proyecto para construir una planta de producción de antivenenos por parte del INSPI con el fin de retomar la producción nacional para satisfacer las necesidades del país.

**México:** Los antivenenos son producidos en el sector público por Laboratorios de Biológicos y Reactivos de México, S.A. de C.V. (BIRMEX), la cual es una empresa 100% propiedad del Gobierno federal. La producción de antivenenos en esta institución se inició en 1930. Produce antivenenos para tratamiento de envenenamientos por escorpiones y por serpientes. El ente regulador de México es la Comisión Federal para la Protección contra Riesgos Sanitarios (COPEFRIS), responsable de analizar y liberar los antivenenos. BIRMEX cuenta con certificado de BPM otorgado por este organismo. Los productos de BIRMEX se distribuyen fundamentalmente en México.

**Perú:** La producción de antivenenos está a cargo del Centro Nacional de Productos Biológicos, que forma parte del Instituto Nacional de Salud (INS), del Ministerio de Salud. Produce tres tipos de antivenenos contra venenos de serpientes y un antiveneno antiaracnídico. Actualmente se efectúan procesos de mejoras en infraestructura y para adecuarse a los requerimientos de la agencia reguladora del Perú.

**Venezuela:** Hace 38 años los antivenenos se producen en Laboratorios BIOTECFAR, empresa pública con capital accionario de la Fundación de la Universidad Central de Venezuela y del Centro de Biotecnología de la Facultad de Farmacia de la misma institución. Produce antiveneno para tratar envenenamientos por mordeduras de serpientes y también antiveneno antiescorpiónico. La producción ha declinado en los últimos cuatro años. Existen actualmente dificultades para la producción continua de antivenenos, las cuales se espera superar mediante inversión en infraestructura y adecuación de procedimientos de adquisiciones.

Informaciones adicionales de los laboratorios y sus productos, con aspectos técnicos específicos de cada uno, se encuentran en los Cuadros 1 a 4.

Los representantes de los laboratorios que atendieron el taller presentaron las necesidades de cooperación y apoyo técnico consensuados que se describen a continuación:

### Investigación y desarrollo

Se plantearon las necesidades para mejorar los esquemas de inmunización, las cuales incluyen el mejoramiento de las colecciones de serpientes y artrópodos; además, se discutió la importancia de la preparación y el control de calidad de los venenos, al tenor de las guías de producción y control de antivenenos de la OMS (19). Se analizó la conveniencia de aplicar la información proteómica e inmunológica que se tiene de venenos con el fin de optimizar las mezclas de inmunización para generar la mayor reactividad cruzada posible. Así mismo, se comentó la relevancia de investigar en el tema de esquemas de inmunización, incluyendo el uso de adyuvantes, para mejorar la respuesta inmune de caballos a los venenos.

**CUADRO 1.** Laboratorios públicos productores de antivenenos de América Latina que participaron en el taller

País	Laboratorio	Tipo de sustancia activa	Cantidad de frascos o ampollas producidos (año o periodo)
Argentina	Instituto Nacional de Producción de Biológicos, ANLIS “Dr. Carlos Malbrán”	F(ab’)_2_	25 000 (2017)
Bolivia	Instituto Nacional de Laboratorios de Salud – INLASA	IgG	16 500 (2018)
Brasil	Instituto Butantan – IB	F(ab’)_2_	502 400 (12 meses 2017-2018)
	Fundação Ezequiel Dias – FUNED	F(ab’)_2_	150 000 a 200 000/ año (promedio histórico)
	Instituto Vital Brazil – IVB	F(ab’)_2_	200 000 /año (promedio histórico)
	Centro de Produção e Pesquisa em Imunobiológicos – CPPI	F(ab’)_2_	5 000 /año (promedio histórico)
Colombia	Instituto Nacional de Salud – INS	IgG	25 000 (2018)
Costa Rica	Instituto Clodomiro Picado – ICP	IgG	130 000 (12 meses 2016-2017)
Ecuador	Instituto Nacional de Investigación en Salud Pública-INSPI- Dr. Leopoldo Izquieta	Datos no disponibles	Datos no disponibles
México	Laboratorios de Biológicos y Reactivos de México – BIRMEX	F(ab’)_2_	300 000 /año (promedio en los últimos 5 años)
Perú	Centro Nacional de Productos Biológicos – Instituto Nacional de Salud – CNPB	IgG	13 610 (2018)
Venezuela	Centro de Biotecnologia, Facultad de Farmacia de la Universidad Central de Venezuela – BIOTECFAR	F(ab’)_2_	31 840 (2018)

***Fuente:*** Reunión de laboratorios públicos productores de antivenenos de América Latina, 23-25 de octubre del 2018, Instituto Butantan, São Paulo, Brasil

F(ab’)_2_: fragmento de IgG resultante de la digestión enzimática por pepsina

Igualmente se examinó la necesidad de mejorar en algunos laboratorios los procesos de fraccionamiento de plasma hiperinmune, y también la necesidad de cooperación para implementar y validar métodos de remoción e inactivación de virus durante el proceso de fraccionamiento del plasma hiperinmune. Representantes de varios laboratorios plantearon el interés por introducir la liofilización en la producción de antivenenos. Además, se analizó la conveniencia de desarrollar métodos *in vitro*, que permitan reducir el uso de animales en la prueba de pirógenos. Para llevar adelante estos procesos es fundamental el establecimiento de vínculos de cooperación científica y técnica permanentes entre los laboratorios productores y de control de calidad de la región, y entre estos y los grupos de investigación de instituciones universitarias y otros institutos de investigación, discusiones semejantes han sido conducidas en diferentes países de la región, llamando la atención sobre la necesidad de estructurar cooperaciones técnicas (20–24).

Un ejemplo de cooperación regional es el caso de los antivenenos anti-*Micrurus*, los cuales son difíciles de producir debido a la dificultad para obtener estos venenos, se planteó la importancia de promover proyectos similares al efectuado en la década de 1970, cuando se produjo un antiveneno anti-*Micrurus* polivalente mediante cooperación regional con apoyo de la OPS (25).

### Estudios preclínicos de eficacia de antivenenos

El conocimiento del perfil de la capacidad neutralizante de los antivenenos contra diferentes venenos es una tarea central en la región latinoamericana. Una serie de proyectos desarrollados a niveles nacional y regional han generado un robusto cuerpo de conocimientos sobre el perfil neutralizante de los antivenenos en fase preclínica (26–30). Estos estudios han mostrado que algunos antivenenos de la región tienen un amplio perfil neutralizante contra venenos de serpientes de la familia Viperidae, en tanto en otros casos la cobertura de neutralización cruzada contra venenos heterólogos es más limitada (26). Es necesario extender estas investigaciones para cubrir otros antivenenos cuyo perfil neutralizante no ha sido debidamente estudiado, siguiendo el modelo del estudio efectuado en el proyecto apoyado por CYTED, en el cual participaron diez laboratorios de siete países de la región (31). Es deseable que las conclusiones de dichos estudios apoyen la adquisición de antivenenos de países que no cuentan con laboratorios productores (como Paraguay, Uruguay, las Guyanas y algunos países del Caribe), o en situaciones en las que el laboratorio nacional no produzca un antiveneno específico o la cantidad producida sea insuficiente para la demanda.

### Fortalecimiento de los laboratorios públicos

El cumplimiento de los requerimientos internacionales para laboratorios productores conlleva la urgente necesidad de invertir en diversos aspectos de infraestructura, equipamiento, validación de procesos y otros aspectos. Estas necesidades demandan apoyo económico y de otros tipos por parte de las dirigencias de las instituciones y de los ministerios de salud. En algunos países, la suspensión de las actividades en laboratorios productores, debido al incumplimiento de las BPM, ha llevado a una reducción de la disponibilidad de estos productos, con el consecuente impacto en la salud pública. Esta situación debe atenderse con urgencia por parte de las autoridades de los institutos y de los ministerios. Cuando se analiza la situación descrita hace cinco años, es evidente que las dificultades para cumplir integralmente con las normas de BPM ya estaban presentes (32). Desde entonces, la respuesta de los diversos laboratorios ha sido distinta, mientras algunos laboratorios alcanzaron la certificación de BPM, otros tuvieron dificultades para lograrlo y, como consecuencia, debieron disminuir o interrumpir sus actividades de producción.

**CUADRO 2.** Antivenenos producidos por laboratorios públicos de América Latina contra venenos de serpientes de los géneros *Bothrops* y *Lachesis*

Laboratorio	Producto	Venenos utilizados en la inmunización	Capacidad neutralizante mínima mg veneno/mL de antiveneno
ANLIS	Antiveneno bivalente (líquido)	Balt Bdip	2,5 mg *Balt* 1,5 mg *Bdip*
	Antiveneno tetravalente (líquido)	Balt Bdip Bjar Bjussu	2,5 mg *Balt* 1,5 mg *Bdip* y *Bjar* 1,8 mg *Bjussu*
INLASA	Suero antiofídico botrópico-crotálico (líquido)	Bnbo Cdter	1,5 mg *Bnbo* 0,5 mg *Cdter*
	Suero antiofídico botrópico-laquésico (líquido)	Bothrops Lachesis	2,5 mg *Bnbo* 2,5 mg *Lmut*
IB, FUNED, IVB, CPPI	Suero antibotrópico (líquido)	Bjar Balt Bjussu Bmoo Bneu	5 mg *Bjar*
IB, FUNED, IVB	Suero antibotrópico-crotálico (líquido)	Bjar Balt Bjussu *Bmoo Bneu* + Cdter Cdcol	5 mg *Bjar* 1,5 mg *Cdter*
IB, FUNED	Suero antibotrópico-laquético (líquido)	Bjar Balt Bjussu *Bmoo Bneu* + Lmut	5 mg *Bjar* 3 mg *Lmut*
INS	Suero antiofídico polivalente (líquido)	Basp Batr Cdu Lmut Lach Plan	7 mg *Bothrops* 1 mg *Crotalus* 1,5 mg *Lmut* 6 mg *Lach* 4 mg *Plan*
ICP	Suero antiofídico polivalente PoliVal-ICP (líquido y liofilizado)	Basp Csim Lste	3 mg *Basp* 2 mg *Csim* 3 mg *Lste*
BIRMEX	Faboterápico polivalente antiviperino (liofilizado)	Basp Cbas	79 DL_50_ *Cbas* y 78 DL_50_ *Basp*
CNPB	Suero antibotrópico polivalente (líquido)	Batr Bbra Bpic Bbar Bhyo	2,5 mg *Batr*
BIOTECFAR	Suero antiofídico polivalente (líquido)	Bcol Cdcum	2 mg *Bcol* y 1,5 mg *Cdcum*

***Bothrops spp*:***Balt= B alternatus; Bdip= B. diporus, Bjar= B. jararaca; Bjus= B. jararacussu; Bmoo= B. moojeni; Bneu= B. neuwiedi; Basp= B. asper; Batr= B. atrox; Bbra= B. brazili; Bpic= B. pictus; Bbar= B. barnetti; Bcol= B. colombiensis*

***Bothrops ssp*:***Bnbo= B. neuwiedi bolivianus*

***Bothrocophias sp*:***Bhyo= B. hyoprora*

***Crotalus****spp: Csim= C. simus; Cdu= C. durissus; Cbas= C. basilicus*

***Crotalus durissus ssp*:***Cdter= C.d. terrificus; Cdcol= C. d. collilineatus; Cdcum= C. d. cumanensis;*

***Lachesis sp*:***Lmut= Lachesis muta; Lach= L. acrochorda; Lste= L. stenophrys*

***Porthidium sp*:***Plan= P. lansbergii*

DL_50_: Dosis Letal Media

ANLIS: Instituto Nacional de Producción de Biológicos, ANLIS “Dr. Carlos Malbrán” (Argentina); INLASA: Instituto Nacional de Laboratorios de Salud (Bolívia); IB: Instituto Butantan (Brasil); FUNED: Fundação Ezequiel Dias (Brasil); IVB: Instituto Vital Brazil (Brasil); CPPI: Centro de Produção e Pesquisa de Imunobiológicos (Brasil); INS: Instituto Nacional de Salud (Colombia); ICP: Instituto Clodomiro Picado (Costa Rica); BIRMEX: Laboratorios de Biológicos y Reactivos de México (México); CNPB: Centro Nacional de Productos Biológicos (Perú); BIOTECFAR: Centro de Biotecnologia, Facultad de Farmacia de la Universidad Central de Venezuela (Venezuela)

Existe una importante tradición de producción de antivenenos en América Latina, que data de los esfuerzos pioneros de inicios del siglo XX. Este universo productivo requiere ser fortalecido, para lo que se requiere concebir esquemas de financiamiento novedosos y creativos por parte de los gobiernos, para garantizar la continuidad de las actividades de los laboratorios productores y la disponibilidad y accesibilidad de estos productos a la población (27, 28, 32). Estos laboratorios son instituciones públicas que desde hace décadas vienen contribuyendo para la solución de problemas de salud pública, constituyendo verdaderos patrimonios nacionales científicos y tecnológicos.

### Capacitación de personal técnico y profesional

En el taller se planteó la necesidad de desarrollar programas permanentes de entrenamientos específicos dirigidos a técnicos y profesionales de los laboratorios productores y de control de calidad de antivenenos en temas de investigación, desarrollo, y estandarización de procesos y métodos. Los avances en nuevas metodologías de producción y análisis deben ser igualmente compartidas en la región, de acuerdo con los intereses y capacidades de cada laboratorio. Este tipo de actividades han sido desarrolladas en el pasado, con el auspicio de organizaciones como la *Japan International Cooperation Agency* (JICA), la red Ciencia y Tecnología para el Desarrollo (CYTED) (28, 29) y el fondo FEMCIDI de la Organización de Estados Americanos (32).

### El papel de la Cooperación Técnica Regional de OPS/OMS a través de PANAFTOSA

El taller permitió conocer las debilidades debilidades y las fortalezas en los laboratorios públicos productores de antivenenos en América Latina y analizar las necesidades de las diferentes instituciones, lo cual sirvió para abrir espacios de cooperación entre países y grupos. Se hizo evidente la importancia de trabajar de manera integrada en la región, para fomentar las fortalezas y superar las debilidades. En este contexto, se consideró esencial el papel que debe desempeñar la OPS, a través de PANAFTOSA, en la promoción de actividades regionales diversas dirigidas al cumplimiento de las metas planteadas en la resolución de la Asamblea Mundial de la Salud y en el plan estratégico de la OMS. Se creó una Red de Cooperación de Laboratorios Públicos Productores de Antivenenos de América Latina (RELAPA), bajo la coordinación de PANAFTOSA, con el propósito de impulsar el intercambio de informaciones y la cooperación entre laboratorios. Se enfatizó en la importancia de desarrollar esfuerzos conjuntos y procesos coordinados para estudiar la viabilidad de la creación de depósitos regionales y esquemas de distribución efectivos, idealmente bajo la coordinación de OPS y asumidos por los países. De esa manera, se pretende ampliar la disponibilidad y accesibilidad, incluyendo a países que no poseen sus propios laboratorios productores nacionales, como Paraguay, varios países de Centroamérica y Caribe, o aquellos en que la producción de determinados antivenenos por un laboratorio no sea suficiente para atender la demanda nacional. Así mismo, se concluyó que los ministerios de salud deben jugar un papel protagónico en el impulso de estos procesos y garantizar la disponibilidad y acceso oportuno de antivenenos en los diversos niveles de atención.

**CUADRO 3.** Antivenenos producidos por laboratorios públicos de América Latina contra venenos de serpientes de los géneros *Crotalus* y *Micrurus*

Laboratorio	Producto	Venenos utilizados en la inmunización	Capacidad neutralizante mínima mg veneno/mL de antiveneno
ANLIS	Antiveneno crotálico (líquido)	Cdter	1,0 mg *Cdter*
	Antiveneno *Micrurus* (líquido)	Mpyr	1,0 mg *Mpyr*
INLASA	Suero antiofídico botrópico-crotálico (líquido)	Bnbo Cdter	1,5 mg *Bnbo* 0,5 mg *Cdter*
IB, FUNED, IVB, CPPI	Suero anticrotálico (líquido)	Cdter Cdcol	1,5 mg *Cdter*
IB, FUNED, IVB	Suero antibotrópico-crotálico (líquido)	Bjar Balt Bjussu Bmoo Bneu Cdter Cdcol	5 mg *Bjar* 1,5 mg *Cdter*
IB, FUNED	Suero antielapídico	*Mfro, Mcor*	*1,5 mg Mfro*
INS	Suero antiofídico polivalente (líquido)	Basp Batr Cdu Lmut Lach Plan	7 mg *Bothrops* 1 mg *Crotalus* 1,5 mg *Lmut* 6 mg *Lach* 4 mg *Plan*
ICP	Suero antiofídico polivalente PoliVal-ICP (líquido y liofilizado)	B.asper Csim Lste	3 mg *B. asper* 2 mg *Csim* 3 mg *Lste*
	Suero antiofídico anticoral (líquido)	Mnig	0,3 mg *Mnig* 0,3 mg *Mcar* 0,125 mg *Mful*
BIRMEX	Faboterápico polivalente Antiviperino (liofilizado)	B. asper Cbas	79 DL_50_ *Cbas* 78 DL_50_ *Basp*
CNPB	Suero anticrotálico monovalente (líquido)	Cdter	1,5 mg *Cdter*
	Suero antilachésico monovalente (líquido)	L. muta	2,5 mg *L. muta*
BIOTECFAR	Suero antiofídico polivalente (líquido)	Bcol Cdcum	2 mg *Bcol* 1,5 mg *Cdcum*

***Bothrops spp*:***Balt= B alternatus; Bdip= B. diporus, Bjar= B. jararaca; Bjus= B. jararacussu; Bmoo= B. moojeni; Bneu= B. neuwiedi; Basp= B. asper; Batr= B. atrox; Bbra= B. brazili; Bpic= B. pictus; Bbar= B. barnetti; Bcol= B. colombiensis*

***Bothrops ssp*:***Bnbo= B. neuwiedi bolivianus*

***Bothrocophias sp*:***Bhyo= B. hyoprora*

***Crotalus****spp: Csim= C. simus; Cdu= C. durissus; Cbas= C. basilicus*

***Crotalus durissus ssp*:***Cdter= C.d. terrificus; Cdcol= C. d. collilineatus; Cdcum= C. d. cumanensis;*

***Lachesis sp*:***Lmut= Lachesis muta; Lach= L. acrochorda; Lste= L. stenophrys*

***Porthidium sp*:***Plan= P. lansbergii*

***Micrurus*spp**: *Mpyr* = *Micrurus pyrrhocryptus, Mnig= M. nigrocinctus, Mcar= M. carinicaudus, Mful= M. fulvius, Mfro= M. frontalis, Mcor= M. corallinus*

DL_50_: Dosis Letal Media

ANLIS: Instituto Nacional de Producción de Biológicos, ANLIS “Dr. Carlos Malbrán” (Argentina); INLASA: Instituto Nacional de Laboratorios de Salud (Bolívia); IB: Instituto Butantan (Brasil); FUNED: Fundação Ezequiel Dias (Brasil); IVB: Instituto Vital Brazil (Brasil); CPPI: Centro de Produção e Pesquisa de Imunobiológicos (Brasil); INS: Instituto Nacional de Salud (Colombia); ICP: Instituto Clodomiro Picado (Costa Rica); BIRMEX: Laboratorios de Biológicos y Reactivos de México (México); CNPB: Centro Nacional de Productos Biológicos (Perú); BIOTECFAR: Centro de Biotecnologia, Facultad de Farmacia de la Universidad Central de Venezuela (Venezuela)

**CUADRO 4:** Antivenenos producidos por laboratorios públicos de América Latina contra venenos de artrópodos

Laboratorio	Producto	Venenos utilizados en la inmunización	Capacidad neutralizante mínima por mL de antiveneno
ANLIS	Antiveneno *Latrodectus* (líquido)	Latrodectus sp	1000 DL_50_ *Latrodectus* sp
	Antiveneno *Phoneutria* (líquido)	Phoneutria nigriventer	1,5 DMM *P. nigriventer*
	Antiveneno *Loxoceles* (líquido)	Loxosceles laeta	190 DMN *L. laeta*
	Antiveneno escorpión (líquido)	Tityus trivittatus	25 DL_50_ *T. trivittatus*
INLASA	Suero antilatrodectus (líquido)	Latrodectus	2500 DL_50_ *Latrodectus* sp
IB, FUNED, IVB	Suero antiescorpiónico (líquido)	T. serrulatus	1,0 mg *T. serrulatus*
IB	Suero antiracnídico (líquido)	L. gaucho P. nigriventer T. serrulatus	1,5 DMM *T. serrulatus*, 1,5 DMM *P. nigriventer* 15 DMN *L. gaucho*
	Suero antilonómico (líquido)	Lonomia obliqua	3,5 mg *L. obliqua*
INS	Antiveneno lonómico polivalente (líquido)	L. casanarensis L. orientoandensis	0,35 mg *L. casanarensis*
BIRMEX	Faboterápico polivalente Antialacrán (liofilizado)	Centruroides limpidus limpidus C. suffusus suffusus C. noxius	30 DL_50_ *Centruroides* sp
CNPB	Suero antiloxoscélico monovalente (líquido)	L. laeta	80 glándulas de *L. laeta*
BIOTECFAR	Suero antiescorpiónico (líquido)	Tityus	0,2 mg *Tityus*

DMM: Dosis mortal minima

DMN: Dosis Mínima Necrotizante

DL_50_: Dosis Letal Media

ANLIS: Instituto Nacional de Producción de Biológicos, ANLIS “Dr. Carlos Malbrán” (Argentina); INLASA: Instituto Nacional de Laboratorios de Salud (Bolívia); IB: Instituto Butantan (Brasil); FUNED: Fundação Ezequiel Dias (Brasil); IVB: Instituto Vital Brazil (Brasil); CPPI: Centro de Produção e Pesquisa de Imunobiológicos (Brasil); INS: Instituto Nacional de Salud (Colombia); ICP: Instituto Clodomiro Picado (Costa Rica); BIRMEX: Laboratorios de Biológicos y Reactivos de México (México); CNPB: Centro Nacional de Productos Biológicos (Perú); BIOTECFAR: Centro de Biotecnologia, Facultad de Farmacia de la Universidad Central de Venezuela (Venezuela)

### Conclusiones y recomendaciones

Los resultados de ese taller fueron altamente satisfactorios, ya que se logró un diagnóstico de la situación regional en el tema, que permitió detectar los avances y las limitaciones. Se renovaron lazos de cooperación y solidaridad entre los grupos de la región y se espera que las informaciones presentadas, con base en los datos suministrados y en las discusiones consensuadas, se conviertan en una línea de base para RELAPA y para futuras acciones de cooperación regional. Uno de los resultados esperados con la creación de RELAPA es que el intercambio de conocimientos técnicos contribuya para que los laboratorios que necesitan cumplir requisitos regulatorios logren hacerlo, para así retomar la producción de antivenenos para sus países lo antes posible. La disponibilidad de antivenenos de calidad es una condición imprescindible para la organización y ejecución efectiva de los programas de vigilancia y control en cada uno de los países y con eso se logre el objetivo de reducir la morbilidad y mortalidad causada por los envenenamientos por animales ponzoñosos.

Para el logro de sus objetivos, se necesita fortalecer RELAPA como un foro activo de intercambio entre laboratorios productores y de interlocución de estos con los respectivos ministerios de salud y entidades internacionales. Los aspectos específicos de la forma de funcionamiento, lineamientos básicos, líneas de acción, metas y objetivos de corto, mediano y largo plazo de esta red regional serán construidos colectivamente por los integrantes de la misma. Se pretende que RELAPA sea un soporte eficaz para PANAFTOSA/OPS, que tiene la tarea de coordinar las acciones concertadas con el plan estratégico de OMS en nuestra región, especialmente en lo que respecta al incremento de la cantidad disponible de antivenenos y del número de laboratorios productores a nivel global.

## Miembros de la RELAPA:

Los participantes de los laboratorios que integran la Red de Laboratorios Públicos Productores de Antivenenos de América Latina, coautores de este trabajo, son: José Christian Dokmetjian (Instituto Nacional de Producción de Biológicos, ANLIS “Dr. Carlos Malbrán”, Argentina); Gil Patrick Fernandes Coeuillet (Instituto Nacional de Laboratorios de Salud, Bolivia); Fabricio Pettito Assis, José Roberto Marcelino (Instituto Butantan, Brasil); Luis Eduardo Cunha (Instituto Vital Brazil, Brasil); Rômulo Toledo (Fundação Ezequiel Dias, Brasil); Elisa Perez (Centro de Produção e Pesquisa de Imunobiológicos, Brasil); Nestor F. Mondragón G (Instituto Nacional de Salud, Colombia); Alberto Alape-Girón (Instituto Clodomiro Picado, Costa Rica); Ivan Norberto Torres (Instituto Nacional de Investigación en Salud Pública “Dr. Leopoldo Izquieta”, Ecuador); Pedro Garcia Bañuelos (Laboratorios de Biológicos y Reactivos de México, México); Eliana Cecilia Sui Delgado (Centro Nacional de Productos Biológicos Instituto Nacional de Salud, Perú); Mariana Cepeda (Centro de Biotecnología, Facultad de Farmacia de la Universidad Central de Venezuela, Venezuela).

## Agradecimientos.

Se agradece el apoyo brindado por Astrid Meira y Veronica Pereira da Costa en la organización del taller.

## Contribución de los autores.

HWF y JMG elaboraron la primera versión del artículo. Todos los autores analizaron los datos presentados, interpretaron la discusión y revisaron el manuscrito. Todos los autores aprobaron la versión final del texto.

## Financiación.

Fundação Butantan y PANAFTOSA/OPS

## Declaración.

Las opiniones expresadas en este manuscrito son responsabilidad del autor y no reflejan necesariamente los criterios ni la política de la RPSP/ PAJPH y/o de la OPS.
